# *ATM* rs189037 significantly increases the risk of cancer in non-smokers rather than smokers: an updated meta-analysis

**DOI:** 10.1042/BSR20191298

**Published:** 2019-06-28

**Authors:** Xiaoxia He, Peng Wang, Ying Li, Na Shen

**Affiliations:** 1 Department of Laboratory Medicine, Tongji Hospital, Tongji Medical College, Huazhong University of Science and Technology, Wuhan 430030, China; 2 Institute and Department of Infectious Disease, Tongji Hospital, Tongji Medical College, Huazhong University of Science and Technology, Wuhan, China

**Keywords:** Ataxia telangiectasia mutated (ATM), cancer, meta-analysis, rs189037, risk, smoking

## Abstract

Rs189037 (G>A) is an important functional variant with *ataxia telangiectasia mutated* (*ATM*) gene, which might affect *ATM*’s expression involvement in several human cancers. Increasing evidence reveals that smoking-related cancers have distinct molecular characteristics from non-smoking cancers. Until now, the role of *ATM* rs189037 in cancer risk stratified by smoking status still remains unclear. To evaluate the association between *ATM* rs189037 and cancer risk based on smoking status, we performed this meta-analysis by a comprehensive literature search via databases of PubMed, Embase, Web of Science and CNKI, updated till January 2019. Multivariate-adjusted odds ratios (ORs) and 95% confidence intervals (CIs) were extracted from eligible studies if available, to assess the relationship strengths. A total of seven eligible studies were included, comprising 4294 cancer patients (smokers: 1744 [40.6%]) and 4259 controls (smokers: 1418 [33.3%]). Results indicated a significant association of *ATM* rs189037 with cancer risk. In non-smokers, compared with GG genotype, AA genotype increased a 1.40-fold risk of overall cancer (OR = 1.40, 95% CI = 1.15–1.70, *P*_heterogeneity_=0.433, *I^2^* = 0.0%). Subgroup analysis in lung cancer (LC) also exhibited a significant result (OR = 1.41, 95% CI = 1.15–1.73, *P*_heterogeneity_=0.306, *I^2^* = 17.0%) only in non-smokers. However, the association was not observed in smokers, no matter for overall cancer or for LC. Our findings highlight that *ATM* rs189037 significantly increases cancer susceptibility in non-smokers, rather than in smokers. The association is prominent in LC.

## Introduction

The incidence and mortality of human cancer are rapidly increasing, with an estimation of 18.1 million new cases and 9.6 million deaths in 2018 worldwide [1]. It is expected to be the leading cause of death and the single greatest threat to life expectancy in the 21st century [2]. Several environmental factors are revealed to play a role in carcinogenesis, including air pollutants, alcoholism, and virus infection [3,4]. Smoking is estimated to account for more than 30% of all cancer deaths and 90% of lung cancer (LC) deaths, and approximately 62% of all recently diagnosed cancer patients are reported as smokers, which is becoming the most prominent risk factor for human cancer [5].

Besides environmental risk factors, genetic predisposition is also crucial for occurrence and development of cancer [6–8]. Researches have uncovered many candidate genes associated with tumorigenesis, such as DNA damage checkpoint genes (DDCGs). As a famous member of DDCG, *ataxia telangiectasia mutated* (*ATM*) encodes a serine/threonie protein kinase to play a major role in cell cycle checkpoints and DNA repair initiation by phosphorylating some key factors (e.g. p53), which is frequently mutated in human cancers [7,9]. *ATM* rs189037 (G>A), located at the 5′UTR of its promoter, is an important variant reportedly involving susceptibility to several cancers, but results remain inconclusive [10–16].

Some meta-analyses have made efforts to evaluate the role of *ATM* rs189037 in cancer risk. An early one was performed by Kang et al. (2014) [17], which only included one case–control study about rs189037. Then, Bhowmik et al. (2015) [18] and Yan et al. (2017) [19] conducted such analyses respectively focusing on specific cancer types. The latest meta-analysis was published by Zhao et al. (2019) [20], which explored the association between rs189037 and all cancer risk. However, all these meta-analyses did not consider effects of smoking, a most important environmental risk factor affecting most types of cancer. Additionally, they pooled results simply based on genotype information, rather than using confounder-adjusted odds ratio (OR), which possibly induced some bias from original studies. Therefore, we carried out this updated meta-analysis, aiming to use more refined data to clarify the effects of *ATM* rs189037 on cancer risk stratified by smoking status.

## Materials and methods

This meta-analysis was carried out according to the statement of the Preferred Reporting Items for Systematic Reviews and Meta-Analyses (PRISMA) [21].

### Literature search and eligibility criteria

Multiple databases including PubMed, Web of Science, Embase, and CNKI were searched for available relevant studies, without any restriction (updated till January 2019). The search items were used as follows: ‘ATM’, ‘polymorphism’, ‘variant’, ‘cancer’, ‘smoking’, ‘cigarette’ and ‘rs189037’. We also performed manual search by reviewing the reference lists of identified publications for potentially relevant studies.

A study was considered eligible if it met all the following criteria: (i) it was a case–control study to investigate the association between *ATM* rs189037 and cancer risk; (ii) it reported the OR and 95% confidence interval (CI), or provided allele frequency and/or genotype distribution of *ATM* rs189037 in cases and controls; and (iii) it evaluated the effects of *ATM* rs189037 on cancer risk stratified by smoking status. If authors published multiple articles based on the same or overlapping datasets, we chose the study with the largest sample size. Exclusion criteria were as follows: (i) review, meta-analysis, comment, conference abstract, or experimental research; and (ii) articles without healthy controls or with duplication of earlier studies. Two independent authors conducted the literature search and study selection and discrepancy was solved by discussion.

### Data extraction and quality evaluation

Two authors independently extracted the items from each included study, including the first author’s name, publication year, cancer type, country, ethnicity, the number of cases and controls, genotyping methods, and proportion of males and smokers. In addition, multivariate-adjusted OR and 95% CI, genotype distribution, and allele frequency based on smoking status were also recorded from these eligible studies.

The quality of each included study was evaluated by the Newcastle–Ottawa scale (NOS), with scores in a range from ‘0’ to ‘9’ [22]. Quality evaluation was not an exclusion criterion for eligible studies (Supplementary Table S1).

### Statistical analysis

The Hardy–Weinberg equilibrium was assessed in genotypes of controls by using a χ^2^ test. The strength of the association between *ATM* rs189037 and cancer risk was measured with OR and 95% CI. Multivariate-adjusted ORs and 95% CIs were preferentially extracted from included studies if available, otherwise unadjusted ORs and 95% CIs were calculated based on genotypes or allele frequencies. Cochran’s Q test and *I^2^* statistic were used to evaluate the heterogeneity among studies, and *P*<0.10 or *I^2^* > 50 % indicates significant heterogeneity [23]. A random-effects model was applied to pool results under significant heterogeneity, otherwise a fixed-effects model was used [24]. Moreover, subgroup analysis was carried out to further explore more specific roles of *ATM* rs189037 in cancer risk. We conducted one-way sensitivity analysis to assess the stability of pooled results. In addition, we also examined publication bias by Begg’s and Egger’s tests [25,26]. A two-sided *P*≤0.05 was considered as significant, unless otherwise specified. Our meta-analysis was performed by Stata 12.0 software (College Station, TX, U.S.A.).

## Results

### Characteristics of included studies

**Figure 1:**
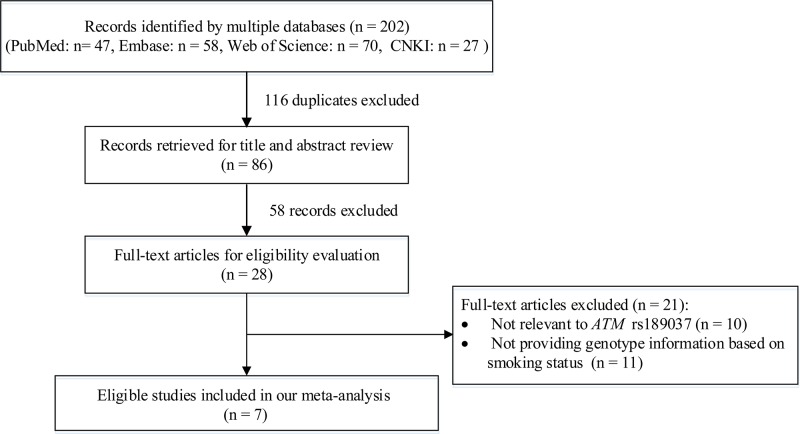
A flowchart of literature search and study selection

Initially, we identified 202 records from a comprehensive search via different databases. After removing 116 duplicates, we also excluded 58 records by reviewing titles and abstracts due to not being original articles (e.g. review, meta-analysis, comment), and not related to cancer risk. Out of the remaining 28 records for full-text review, we further removed 21 studies based on the following reasons: (i) relevant to other variants of *ATM* but not rs189037; and (ii) providing insufficient genotype information of *ATM* rs189037 based on smoking status. Finally, a total of seven eligible studies were included for meta-analysis (Figure 1) [10–16].

Characteristics of these studies are summarized in Table 1. All the subjects were East Asians from China. Overall, there were 2405 males (56.0%) and 1744 smokers (40.6%) in 4294 cancer patients, and were 2331 males (54.7%) and 1418 smokers (33.3%) in 4259 controls. Four studies focused on LC [11–13,15], and the remaining studies focused on oral cancer (OC) [10], esophageal squamous cell carcinoma (ESCC) [14] and colorectal cancer (CRC) [16], respectively. All these studies had an NOS score ≥ 5. Table 2 shows the genotype distribution and allele frequency of *ATM* rs189037 in smokers, non-smokers, and overall subjects.

**Table 1:** Characteristics of included studies

Study	Type	Country	Ethnicity	Cases/Controls	Male (case/control), *n* (%)	Smokers (case/control), *n* (%)	Genotyping method	NOS score
Bau et al. (2010)	OC	China	East Asians	620/620	586 (94.5)/582 (93.9)	458 (73.9)/443 (71.5)	PCR-RFLP	5
Lo et al. (2010)	LC	China	East Asians	730/730	384 (52.6)/384 (52.6)	268 (36.7)/268 (36.7)	MassARRAY	5
Liu et al. (2014)	LC	China	East Asians	852/852	485 (56.9)/490 (57.5)	477 (66.0)/273 (32.0)	TaqMan assay	6
Shen et al. (2014)	LC	China	East Asians	487/516	All females	All non-smokers	TaqMan assay	7
Yu et al. (2015)	ESCC	China	East Asians	303/304	258 (85.1)/253 (83.2)	214 (70.60)/153 (50.3)	TaqMan assay	6
Han et al. (2017)	LC	China	East Asians	181/181	61 (33.7)/61 (33.7)	All non-smokers	MassARRAY	5
Wang et al. (2018)	CRC	China	East Asians	1121/1056	631 (56.3)/561 (53.1)	327 (29.2)/281 (26.6)	TaqMan assay	6

Abbreviation: PCR-RFLP, polymerase chain reaction and restriction fragment length polymorphism.

**Table 2:** Genotype distribution and allele frequency of *ATM* rs189037 stratified by smoking status

Study	Smoking exposure	Genotype (GG/GA/AA)		Minor allele frequency (A allele)		*P_HWE_*
		Cases	Controls	Cases (%)	Controls (%)	
Bau et al. (2010)	Overall	181/277/162	239/285/96	48.47	38.47	0.470
	Smokers	337/121^1^	374/69^1^	-	-	-
	Non-smokers	121/41^1^	150/27^1^	-	-	-
Lo et al. (2010)	Overall	238/345/145	239/354/124	43.61	41.98	0.717
	Smokers	103/122/42	82/131/49	38.58	43.70	0.794
	Non-smokers	135/223/103	157/223/72	46.53	40.60	0.626
Liu et al. (2014)	Overall	217/435/200	264/434/154	49.00	43.54	0.293
	Smokers	120/249/108	87/129/57	48.74	44.51	0.473
	Non-smokers	97/186/92	177/305/97	49.33	43.09	0.075
Shen et al. (2014)	Overall (non-smokers)	148/240/99	152/272/92	44.97	44.19	0.119
Yu et al. (2015)	Overall	106/139/58	114/145/45	42.08	38.65	0.920
	Smokers	72/97/45	59/67/27	43.69	39.54	0.298
	Non-smokers	34/42/13	55/78/18	38.20	37.75	0.223
Han et al. (2017)	Overall (non-smokers)	56/83/39	54/92/32	45.22	43.82	0.507
Wang et al. (2018)	Overall	336/543/227	362/491/191	45.07	41.81	0.280
	Smokers	107/213^2^	106/171^2^	-	-	-
	Non-smokers	229/557^2^	256/511^2^	-	-	-

1 indicates the number of (GG+GA)/AA.

2 indicates the number of GG/(GA+AA).

### Association between *ATM* rs189037 and cancer risk stratified by smoking status

The allelic, dominant, recessive, and codominant models were applied to pool results (Table 3). Overall, *ATM* rs189037 A allele exhibited a 1.17-fold increased risk of cancer compared with the G allele (OR = 1.17, 95% CI = 1.0–1.30). Other genetic models showed the same results. Analysis based on smoking status, we further found a consistent and significant association of *ATM* rs189037 with cancer risk in non-smokers (allelic model: OR = 1.16, 95% CI = 1.05–1.28; dominant model: OR = 1.43, 95% CI = 1.22–1.69; recessive model: OR = 1.14, 95% CI = 1.01–1.29; codominant AA vs GG model: OR = 1.40, 95% CI = 1.15–1.70; additive model: OR = 1.16, 95% CI = 1.06–1.28). Interesting, however, the association was not observed in smokers (all *P*>0.05).

**Table 3:** Meta-analysis for the association between ATM rs189037 and cancer risk stratified by smoking status

Genetic model^1^	Effect size		Heterogeneity		Publication bias	
	*n*	OR (95% CI)	*P*_heterogeneity_	*I^2^*(%)	*P*_Begg_	P_Egger_
Allelic model						
Overall	7	**1.17 (1.06–1.30)**	0.026	58.0	0.764	0.738
Non-smokers	5	**1.16 (1.05–1.28)**	0.324	14.2	0.806	0.514
Smokers	3	1.04 (0.81–1.34)	0.044	68.0	0.956	0.602
Dominant model						
Overall	7	**1.32 (1.19–1.47)**	0.130	39.2	0.548	0.780
Non-smokers	6	**1.43 (1.22–1.69)**	0.642	0.0	0.707	0.894
Smokers	4	1.24 (0.84–1.82)	0.013	72.1	0.734	0.403
Recessive model						
Overall	7	**1.19 (1.08–1.30)**	0.107	42.5	0.548	0.344
Non-smokers	6	**1.14 (1.01–1.29)**	0.504	0.0	0.851	0.263
Smokers	4	1.12 (0.83–1.50)	0.048	62.0	0.308	0.902
Codominant model (AA vs. GG)						
Overall	7	**1.42 (1.18–1.70)**	0.077	47.4	0.881	0.727
Non-smokers	5	**1.40 (1.15–1.70)**	0.433	0.0	1.000	0.608
Smokers	3	1.12 (0.68–1.83)	0.064	63.6	0.602	0.983
Codominant model (GA vs. GG)						
Overall	7	1.11 (1.00–1.22)	0.338	11.9	0.548	0.153
Non-smokers	5	1.01 (0.86–1.18)	0.713	0.0	1.000	0.391
Smokers	3	1.06 (0.72–1.57)	0.063	63.8	0.602	0.852
Additive model						
Overall	7	**1.17 (1.07–1.29)**	0.044	53.6	0.764	0.655
Non-smokers	5	**1.16 (1.06–1.28)**	0.317	15.3	0.806	0.515
Smokers	3	1.04 (0.81–1.34)	0.046	67.5	0.602	0.937

1 Allelic model refers to A allele vs. G allele; dominant model refers to AA+GA vs. GG; recessive model refers to AA vs. GG+GA.

**Figure 2:**
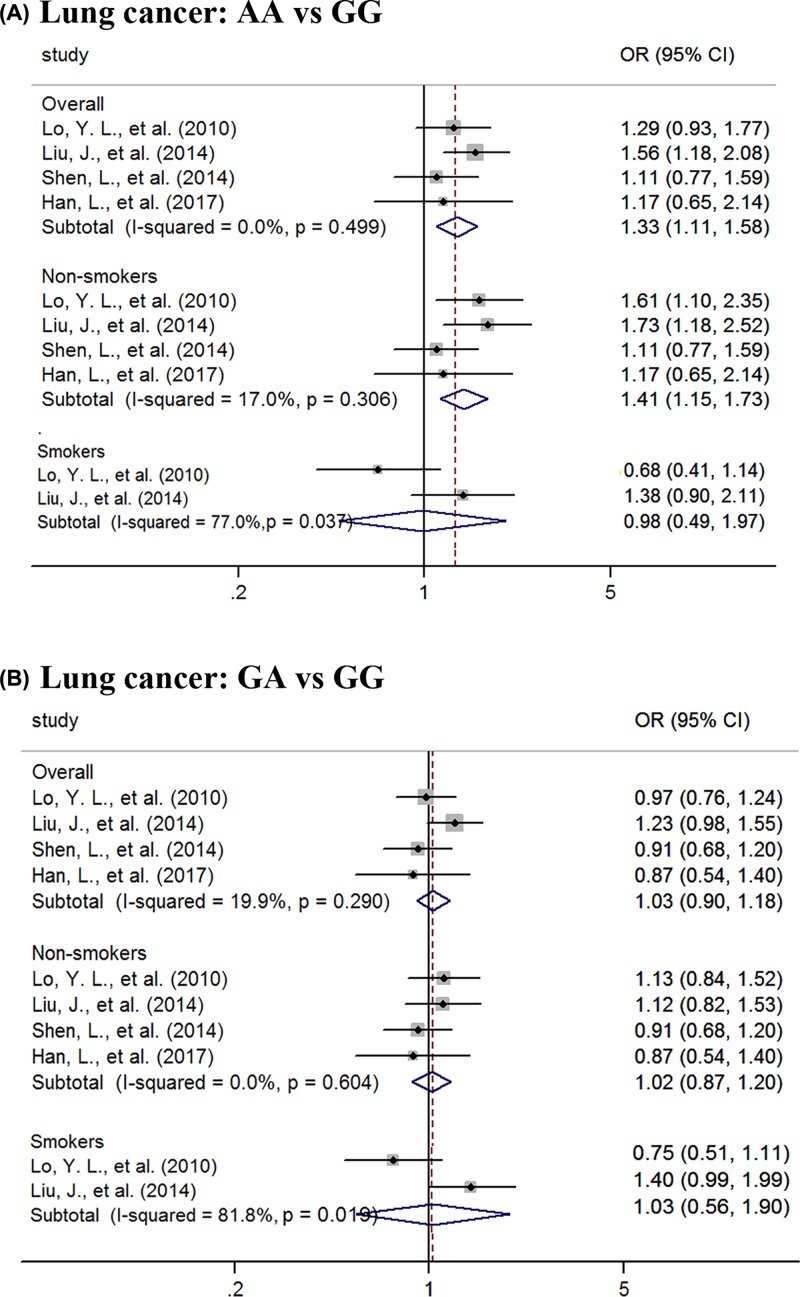
Forest plots of the association between ATM rs189037 and LC risk Forest plots for evaluation of the association between *ATM* rs189037 and LC risk under the codominant models of AA vs GG (**A**) and GA vs GG (**B**).

Considering that smoking is the crucial pathogenic factor for LC, we further evaluated effects of *ATM* rs189037 on LC based on smoking status (Figure 2). Overall, *ATM* rs189037 AA carriers had more risk of LC than wild-type carriers (OR = 1.33, 95% CI = 1.11–1.58). Specially, the association was more notable in non-smokers (OR = 1.41, 95% CI = 1.15–1.73). Also, we did not find any association of this variant with LC risk in smokers. In addition, individuals with GA genotype did not suffer more susceptibility to overall cancer or LC than those with GG genotype, no matter in smokers or in non-smokers (all *P*>0.05).

### Sensitivity analysis and publication bias

To assess the stability of pooled results, we conducted one-way sensitivity analysis by excluding one study at a time. It demonstrated that our pooed results were quite stable in both non-smokers and smokers (Supplementary Figure S1). In addition, results from Begg’s and Egger’s tests showed that there was no obvious publication bias in our meta-analyses under all genetic models (Table 3).

## Discussion

In this meta-analysis, we have four findings as follows: (i) *ATM* rs189037 significantly increased the overall risk of cancer under most of genetic models; (ii) the risky role of *ATM* rs189037 was prominent in non-smokers, but not observed in smokers; (iii) results focusing on LC were consistent with results of overall cancer; and (iv) GA genotype carriers of *ATM* rs189037 appeared not to suffer more cancer risk than GG wild-type carriers, no matter in smokers or in non-smokers.

In agreement with previous meta-analyses [17–20], our work suggested that *ATM* rs189037 is a risky variant for cancer susceptibility, but we have some new highlights. First, we elucidated the association between this variant and cancer risk stratified by smoking status, and specially focused on LC. Results demonstrated different effects of *ATM* rs189037 in non-smokers and in smokers. Second, we pooled results by extracting multivariate-adjusted ORs and 95% CIs if available, greatly reducing confounder bias from original studies. Third, we performed a comprehensive literature and included a study ignored by previous meta-analyses [14].

Rs189037 (G>A) is located at the promoter region of *ATM* gene, alleles of which may possibly have different binding affinities to transcription factors (e.g. AP-2α) or change *ATM* folding structure to affect its mRNA expression, and AA genotype was reported to show a lower *ATM* expression than GG genotype [16,27,28]. Reduced *ATM* expression may impair its normal function, lead to uncontrolled cell cycle, abnormal DNA repair and apoptosis, and finally increase the susceptibility to cancer. This could well explain our findings that individuals with *ATM* rs189037 suffer more cancer risk. Furthermore, our results demonstrated the association was significant in non-smokers rather than in smokers, no matter for overall cancer or for LC. As the most important risk factor for human cancer, smoking induces a serious of potential carcinogens to generate DNA damage and oxidative stress, resulting in gene mutations and genomic instability. Smoking-related cancers have a high mutational load and highly significant molecular heterogeneity [29]. Compared with non-smokers, smokers probably alert more gene pathways to remove those tobacco-induced DNA adducts and activate more antioxidant mechanisms to fight against smoking-related stress. Consistently, we identified obvious heterogeneity among included studies in smokers, whereas we observed a good homogeneity among studies in non-smokers, both for overall cancer and LC (all *P*_heterogeneity_>0.10, *I^2^* < 50%). These results suggest that smoking-related cancers has distinct molecular characteristics from non-smoking-related cancers. Supportively, a recent study revealed that LCr in smokers and in non-smokers showed quite different tumor immune microenvironments [30].

However, some limitations should be acknowledged here. First, only seven studies were eligible for inclusion in our study. The small number of studies possibly affected the conclusion’s extrapolation to some extent. More studies are still needed to verify our results. Second, we did not evaluate the effects of this variant in other ethnic populations, since all included subjects were Chinese. At last, except for LC, number of studies focusing on other cancer is limited, so we could not explore its role based on subgroup analysis in other cancer types.

## Conclusions

Our study highlights that there is a significant association between *ATM* rs189037 and cancer risk in non-smokers, rather than in smokers. This association is prominent in LC. Our work not only provides a new insight into the pathogenic role of *ATM* variants in occurrence of cancer, but also supports the distinct molecular characteristics of cancers between smokers and non-smokers. More studies are still needed to verify our results in the future.

## Availability of data and material

All data in the present study are included in this published article and the additional files.

## Supporting information

**Supplementary Figure S1:** 

**Supplemental Table S1:** Quality evaluation of included studies by Newcastle-Ottawa Scale

## Abbreviations

ATM: *ataxia telangiectasia mutated*
CI: confidence interval
DDCG: DNA damage checkpoint gene
LC: lung cancer
NOS: Newcastle–Ottawa scale
OR: odds ratio
