# Supplementary material for: ATM rs189037 significantly increases the risk of cancer in non-smokers rather than smokers: an updated meta-analysis
Source: Biosci Rep. 2019 Jun 28;39(6):BSR20191298. doi: 10.1042/BSR20191298 (PMC6597848; doi:10.1042/BSR20191298)
Supplement: Supplementary file 1 [file bsr20191298_Supp1.pdf]

GA vs GG: Non-smokers

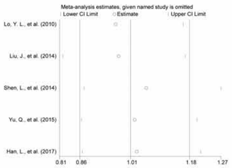

GA vs GG: Smokers

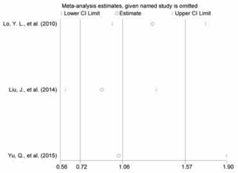

AA vs GG: Non-smokers

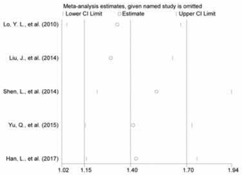

AA vs GG: Smokers

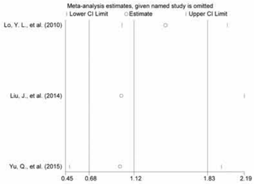

Additive model: Non-smokers

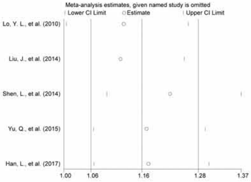

Additive model: Smokers

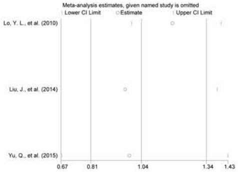

Recessive model: Non-smokers

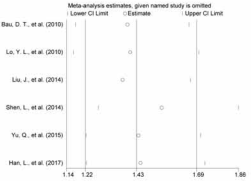

Recessive model: Smokers

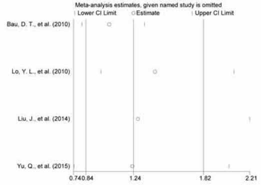

Dominant model: Non-smokers

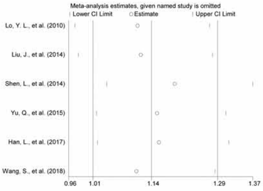

Dominant model: Smokers

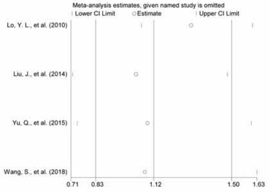

Allelic model: Non-smokers

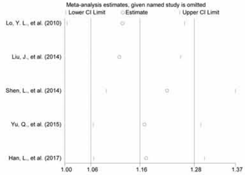

Allelic model: Smokers

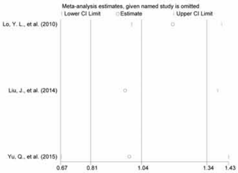

Supplementary Table S1. Quality evaluation of included studies by Newcastle-Ottawa Scale

Study	Selection (0-4)				Comparability (0-2)		Exposure (0-3)			Score
	CDA	RC	SC	DC	SCEA	AF	AE	SM	NR	
Bau, D. T., et al. (2010)	0	0	0	0	1	1	1	1	1	5
Lo, Y. L., et al. (2010)	1	0	0	0	1	1	1	1	0	5
Liu, J., et al. (2014)	1	0	0	0	1	1	1	1	1	6
Shen, L., et al. (2014)	1	0	0	1	1	1	1	1	1	7
Yu, Q., et al. (2015)	1	0	0	0	1	1	1	1	1	6
Han, L., et al. (2017)	1	0	0	1	1	0	1	1	0	5
Wang, S., et al. (2018)	1	0	0	0	1	1	1	1	1	6

Abbreviations: CDA, case definition adequate; RC, representativeness of the cases; SC, selection of controls; DC, definition of controls; SCEA, study controls for sex and age; AF, study controls for any additional factor; AE, ascertainment of exposure; SM, same method of ascertainment for cases and controls; NR, non-response rate.
